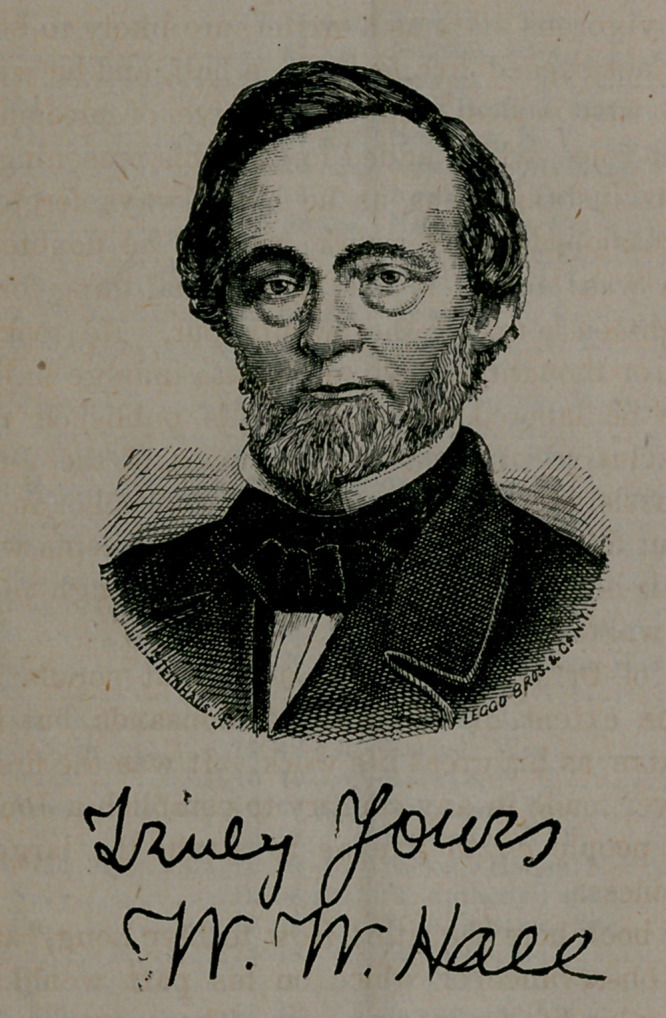# Portrait—Dr. W. W. Hall

**Published:** 1876-06

**Authors:** 


Truly Yours
W. W. Hall

## Figures and Tables

**Figure f1:**